# Supplementary material for: Characterization of Terpenoids from the Ambrosia Beetle Symbiont and Laurel Wilt Pathogen Harringtonia lauricola
Source: J Fungi (Basel). 2023 Dec 7;9(12):1175. doi: 10.3390/jof9121175 (PMC10744799; doi:10.3390/jof9121175)
Supplement: Supplementary file 1 [file jof-09-01175-s001.zip › jof-2720139-supplementary.pdf]

Supplementary Material

Characterization of Terpenoids from the Ambrosia Beetle Symbiont and Laurel Wilt Pathogen *Harringtonia lauricola*

Zhiqiang Zhu [1,†], Chenjie Yang [1,†], Nemat O. Keyhani [2], Sen Liu [1], Huili Pu [1], Peisong Jia [3], Dongmei Wu [4], Philip C. Stevenson [5], G. Mandela Fernández-Grandon [5], Jieming Pan [6], Yuxi Chen [1], Xiayu Guan [7,*] and Junzhi Qiu [1,*]

[1] State Key Laboratory of Ecological Pest Control for Fujian and Taiwan Crops, College of Life Sciences, Fujian Agriculture and Forestry University, Fuzhou 350002, China; sw123zzq@126.com (Z.Z.); cjyang0525@126.com (C.Y.); m17633615410@163.com (S.L.); hdpuhuili@163.com (H.P.); liesleyu@163.com (Y.C.)
[2] Department of Biological Sciences, University of Illinois, Chicago, IL 60607, USA; keyhani@uic.edu
[3] Institute of Plant Protection, Xinjiang Academy of Agricultural Sciences, Urumqi 830091, China; jps-fly@163.com
[4] Biotechnology Research Institute, Xinjiang Academy of Agricultural and Reclamation Sciences, Shihezi 832061, China; wdm0999123@sina.com
[5] Natural Resources Institute, University of Greenwich, Chatham Maritime, Kent ME4 4TB, UK; p.c.stevenson@greenwich.ac.uk (P.C.S.); m.fernandez-grandon@greenwich.ac.uk (G.M.F.-G.)
[6] College of Biology & Pharmacy, Yulin Normal University, Yulin 537000, China; jiemingpan@163.com
[7] College of Horticulture, Fujian Agriculture and Forestry University, Fuzhou 350002, China
* Correspondence: 000q021008@fafu.edu.cn (X.G.); junzhiqiu@126.com (J.Q.); Tel.: +86-591-83789367 (J.Q.)
† These authors contributed equally to this work.

S1. Supplementary Data

S1.1. Spectrum data of compounds 1-9

Compound (**1**): manool (white solid)

ESI -MS *m/z*: 291 $[M+H]^+$, $C_{20}H_{34}O$. ^1H NMR (600 MHz, $CDCl_3$) δ_H(ppm): 5.92 (t, *J*=11.1, 1H), 5.21 (d, *J*=10.8, 2H), 4.50 (s, 2H), 2.04-1.96 (m, 1H), 1.93-1.84 (m, 3H), 1.84-1.79 (m, 1H), 1.55 (t, *J*=11.2, 1H), 1.49 (d, *J*=5.6, 1H), 1.40 (dd, *J*=42.9, 14.0, 3H), 1.33-1.28 (m, 1H), 1.27 (s, 3H), 1.23-1.14 (m, 2H), 1.12 (d, *J*=11.4, 1H), 1.05 (dd, *J*=18.2, 8.7, 1H), 1.00-0.90 (m, 1H), 0.87 (s, 3H), 0.81 (s, 3H), 0.67 (s, 3H); ^{13}C NMR (150 MHz, $CDCl_3$) δ_C(ppm): 148.9 (C-8), 145.4 (C-14), 111.5 (C-5), 106.6 (C-16), 73.6 (C-13), 57.2 (C-9), 55.3 (C-5), 42.1 (C-3), 41.4 (C-12), 39.8 (C-10), 38.9 (C-1), 37.9 (C-7), 33.4 (C-18), 33.2 (C-4), 27.9 (C-16), 24.7 (C-6), 21.9 (C-19), 19.5 (C-2), 17.8 (C-11), 14.5 (C-20).

Compound (**2**):18-hydroxy-7-oxolabda-8(9),13(*E*)-dien-15-oic acid (white acicular crystals)

ESI -MS *m/z*:334$[M+H]^+$,$C_{20}H_{30}O_4$. ^1H NMR (600 MHz, $CDCl_3$) δ_H(ppm): 5.85 (t, *J*=1.3, 1H), 3.37 (d, *J*=10.8, 1H), 3.12 (d, *J*=10.8, 1H), 2.60 (dd, *J*=12.5, 9.7, 1H), 2.35 (t, *J*=12.3, 1H), 2.15-2.09 (m, 1H), 2.08 (d, *J*=1.3, 3H), 2.03-1.98 (m, 2H), 1.92-1.82 (m, 4H), 1.72 (t, *J*=1.0, 3H), 1.68-1.51 (m, 4H), 1.05 (s, 3H), 0.89 (s, 3H); ^{13}C NMR (150 MHz, $CDCl_3$) δ_C(ppm): 200.5 (C-7), 170.2 (C-15), 166.8 (C-9), 157.5 (C-13), 129.6 (C-8), 114.5 (C-14), 71.7 (C-19), 45.7 (C-5), 40.3 (C-4), 38.6 (C-12), 37.8 (C-10), 37.1 (C-1), 36.1 (C-6), 35.6 (C-3), 26.4 (C-11), 19.3 (C-18), 19.3 (C-16), 18.3 (C-2), 18.1 (C-20), 11.6 (C-17).

Compound (**3**):7-oxolabda-8(9),13(*Z*)-diene-15,18-dioic acid (white acicular crystals)

ESI -MS *m/z*: 349$[M+H]^+$, $C_{20}H_{29}O_5$. ^1H NMR (600 MHz, $CDCl_3$) δ_H(ppm): 5.79 (t, *J*=1.3, 1H),

3.42 (d, *J*=10.8, 1H), 3.01 (d, *J*=10.8, 1H), 2.60 (dd, *J*=12.5, 9.7, 1H), 2.35 (t, *J*=12.3, 1H), 2.11-2.07 (m, 1H), 2.08 (d, *J*=1.3, 3H), 2.03-1.98 (m, 2H), 1.89-1.81 (m, 4H), 1.72 (t, *J*=1.0, 3H), 1.62-1.51(m, 4H), 1.05 (s, 3H), 0.89 (s, 3H); ^{13}C NMR (150 MHz, $CDCl_3$) δ_C(ppm): 199.8 (C-7),179.5 (C-18), 167.4 (C-15), 165.6 (C-9), 158.4 (C-13), 130.9 (C-8), 115.8 (C-14), 50.3 (C-19), 43.6 (C-5),40.1 (C-4), 39.6 (C-12), 37.3 (C-10), 36.5 (C-1), 35.1 (C-6), 32.2 (C-3), 29.3 (C-11), 19.4 (C-16), 18.1 (C-2), 15.9 (C-20), 11.1 (C-17).

Compound (**4**):3*β*-hydroxy-8(17),13*E*-labdadien-15-oic acid (white powder)

ESI -MS *m/z*: 321 [M+H]$^+$,$C_{20}H_{32}O_3$. ^{1}H NMR (600 MHz,$CDCl_3$) δ_H(ppm): 5.84 (q, *J*=1.1, 1H), 4.85 (dt, *J*=1.9, 0.9, 2H), 3.26 (dd, *J*=10.4, 7.9, 1H), 2.13 (t, *J*=10.6, 2H), 2.05 (d, *J*=1.3, 3H), 1.96-1.91 (m, 1H), 1.89-1.76 (m, 5H), 1.63 (d, *J*=31.3, 2H), 1.51 (s, 1H), 1.42-1.32 (m, 3H), 0.93 (d, *J*=1.5, 3H), 0.87 (d, *J*=1.5, 3H), 0.62 (s, 3H); ^{13}C NMR (150 MHz, $CDCl_3$) δ_C(ppm): 170.5 (C-15), 161.7 (C-13), 148.5 (C-8), 114.9 (C-14), 107.5 (C-17), 78.8 (C-3), 55.0 (C-9), 53.7 (C-5), 39.9 (C-12), 39.3 (C-10), 38.2 (C-4), 36.9 (C-7), 35.7 (C-1), 27.8 (C-18), 27.3 (C-2), 24.4 (C-6), 24.0 (C-11), 23.3 (C-16), 21.3 (C-19), 13.8 (C-20).

Compound (**5**):enantio-labda-8(20),13(*E*)-dien-15,18-dioic acid (white acicular crystals)

ESI -MS *m/z*: 335 [M+H]$^+$,$C_{20}H_{30}O_4$.^{1}H NMR (600 MHz, $CDCl_3$) δ_H(ppm): 5.85 (br s, 1H), 4.83 (dt, *J*=2.0, 1.0, 2H), 2.47-2.39 (m, 1H), 2.36-2.29 (m, 1H), 2.28-2.23 (m, 1H), 2.11 (t, *J*=10.6, 2H), 2.08 (s, 3H), 2.04 -1.96 (m, 1H), 1.82-1.76 (m, 1H), 1.54-1.45 (m, 1H), 1.44-1.33 (m, 4H), 1.22 (d, *J*=1.5, 3H), 1.19 (dt, *J*=9.2, 2.7, 1H), 0.49 (s, 3H); ^{13}C NMR (150 MHz, $CDCl_3$) δ_C (ppm): 182.8 (C-19), 170.6 (C-15), 161.7 (C-13), 148.5 (C-8), 114.9 (C-14), 107.5 (C-17), 55.9 (C-9), 54.2 (C-5), 45.7 (C-4), 41.7 (C-12), 40.1 (C-10), 39.6 (C-1), 39.3 (C-7), 37.4 (C-3), 28.8 (C-6), 25.8 (C-11), 23.3 (C-16), 20.3 (C-2), 19.0 (C-18), 13.8 (C-20).

Compound (**6**):labd-14-en-19-al,8,13-epoxy (white powder)

ESI -MS *m/z*: 305[M+H]$^+$, $C_{20}H_{32}O_2$. ^{1}H NMR (600 MHz, $CDCl_3$) δ_H(ppm): 9.24 (dq, *J*=2.0, 1.0, 1H), 5.87 (tq, *J*=11.0, 1.1, 1H), 5.14 (s, 1H), 5.13 (s, 1H), 2.12 (ddd, *J*=12.4, 9.4, 6.8, 1H), 1.87 (ddd, *J*=12.3, 9.2, 6.6, 1H), 1.77-1.69 (m, 1H), 1.69-1.60 (m, 3H), 1.60-1.52 (m, 2H), 1.50-1.36 (m, 5H), 1.34-1.29 (m, 1H), 1.27-1.25 (m, 6H), 1.24 (d, *J*=1.1, 3H), 1.23-1.17 (m, 1H), 0.84 (s, 3H); ^{13}C NMR (150 MHz, $CDCl_3$) δ_C(ppm): 207.7 (C-18), 147.7 (C-14), 110.1 (C-15), 75.6 (C-8), 73.4 (C-13), 58.1 (C-9), 51.7 (C-5), 48.1 (C-7), 40.9 (C-2), 39.5 (C-10), 39.1 (C-4), 36.0 (C-12), 35.1 (C-16), 31.4 (C-3), 24.3 (C-17), 19.7 (C-19), 19.3 (C-6), 18.4 (C-2), 18.4 (C-6), 15.8 (C-20).

Compound (**7**): 15*α*-hydroxyhop-17(21)-ene (white solid)

ESI-MS *m/z*:427 [M+H]$^+$,$C_{30}H_{50}O$. ^{1}HNMR (600MHz, $CDCl_3$) δ_H(ppm): 3.77 (dd, *J*=11.3, 5.0, 1H), 2.67 (p, *J*=6.9, 1H), 2.47 (dd, *J*=13.5, 5.0, 1H), 2.24 (m, *J*=19.8, 9.8, 7.6, 3.8, 1H), 2.14 (dd, *J*=15.6, 9.4, 1H), 2.03 (t, *J*=11.1, 1H), 1.73-1.68 (m, 3H), 1.67 (dd, *J*=4.3, 1.3, 1H), 1.66-1.58 (m, 5H), 1.54 (d, *J*=13.5, 2H), 1.47-1.35 (m, 7H), 1.27 (d, *J*=9.6, 6H), 1.19-1.12 (m, 1H), 1.09 (d, *J*=11.8, 6H), 1.00 (d, *J*=6.9, 3H), 0.94 (d, *J*=6.9, 3H), 0.87 (d, *J*=3.6, 9H), 0.81 (s, 3H); ^{13}CNMR (150 MHz, $CDCl_3$) δ_C(ppm):138.3 (C-17), 137.2 (C-19), 74.0 (C-15), 56.2(C-4), 51.8 (C-18), 48.8 (C-10), 48.6 (C-11), 47.5 (C-12), 43.9 (C-2), 42.1 (C-9), 41.4 (C-21), 40.7 (C-6), 37.9 (C-5), 37.4 (C-8), 33.4 (C-3), 33.3 (C-16), 31.3 (C-20), 28.1 (C-22), 26.7 (C-23), 24.1 (C-24), 22.2(C-14), 21.9 (C-25), 21.6 (C-26), 19.3 (C-1), 19.1(C-13), 19.0 (C-7), 16.5 (C-27), 9.9 (C-28) .

Compound (**8**): 15*α*-hydroxy-21*α*-*H*-hopane (white solid)

ESI-MS *m/z*:427[M+H]$^+$, $C_{30}H_{50}O$.^{1}H-NMR (600 MHz, $CDCl_3$) δ_H(ppm): 3.92 (dd, *J* = 10.4, 5.0 Hz, 1H), 2.42 (ddd, *J* = 12.4, 5.0, 2.6 Hz, 1H), 2.29 - 2.19 (m, 1H), 2.12 (dddd, *J* = 16.7, 9.9, 3.2, 1.6

Hz, 1H), 1.84 (ddt, J = 11.9, 4.6, 2.3 Hz, 1H), 1.75 (d, J = 2.0 Hz, 3H), 1.69 - 1.66 (m, 1H), 1.41 - 1.34 (m, 3H), 1.34 - 1.30 (m, 1H), 1.30 - 1.26 (m, 1H), 1.24 (dd, J = 12.8, 2.8 Hz, 1H), 1.21 - 1.06 (m, 4H), 1.04 (s, 2H), 1.03 - 0.96 (m, 1H), 0.78 (m, 1H),0.61 (d, J = 1.1 Hz, 2H).^{13}C NMR (150 MHz, $CDCl_3$)δ_C(ppm): 134.3(C-19), 121.3(C-24), 74.3(C-15), 55.9(C-4), 53.3(C-10), 50.8(C-17), 47.2(C-11), 46.9(C-12), 44.5(C-18), 43.7(C-9), 42.1(C-2), 40.5(C-6), 38.8(C-21), 37.7(C-5), 37.0(C-8), 34.1(C-3), 33.5(C-16), 33.3(C-20), 28.8(C-22), 23.7(C-23), 22.9(C-14), 21.7(C-13), 21.0(C-1), 19.5(C-30),19.1(C-29), 18.8(C-7), 17.4(C-25), 16.0(C-18), 14.5(C-26), 11.6(C-27).

Compound (**9**):15α,22-dihydroxyhopane(white solid)

ESI-MS *m/z*: 444 $[M+H]^+$, $C_{30}H_{52}O_2$. ^{1}HNMR (600 MHz, $CDCl_3$)δ_H(ppm):3.84 (dd, 1H), 1.22 (s, 3H), 1.22 (s, 3H), 1.04 (s, 3H), 0.94 (s, 3H), 0.85 (s, 3H), 0.82 (s, 3H), 0.78 (s, 3H), 0.76 (s, 3H); ^{13}CNMR (150 MHz, $CDCl_3$)δ_C(ppm):74.9 (C - 15), 73.8 (C - 22), 55.9 (C - 5), 50.7 (C-9), 50.6 (C - 17), 50.6 (C - 21), 49.1 (C - 13), 47.3 (C-14), 44.3 (C - 8), 43.6 (C-3), 40.5 (C - 1), 37.7 (C-10), 36.9 (C-7), 33.5 (C-23), 33.3 (C-4), 32.6 (C-16), 31.1 (C-30), 28.7 (C-29), 27.0 (C-20), 24.2 (C-12), 21.7 (C-24), 21.0 (C-11), 19.0 (C-6), 18.8 (C-2), 17.5 (C-28), 15.9 (C-25), 15.9 (C-26), 11.9 (C-27).

S2. Supplementary Figures and Tables

S2.1. Supplementary Figures

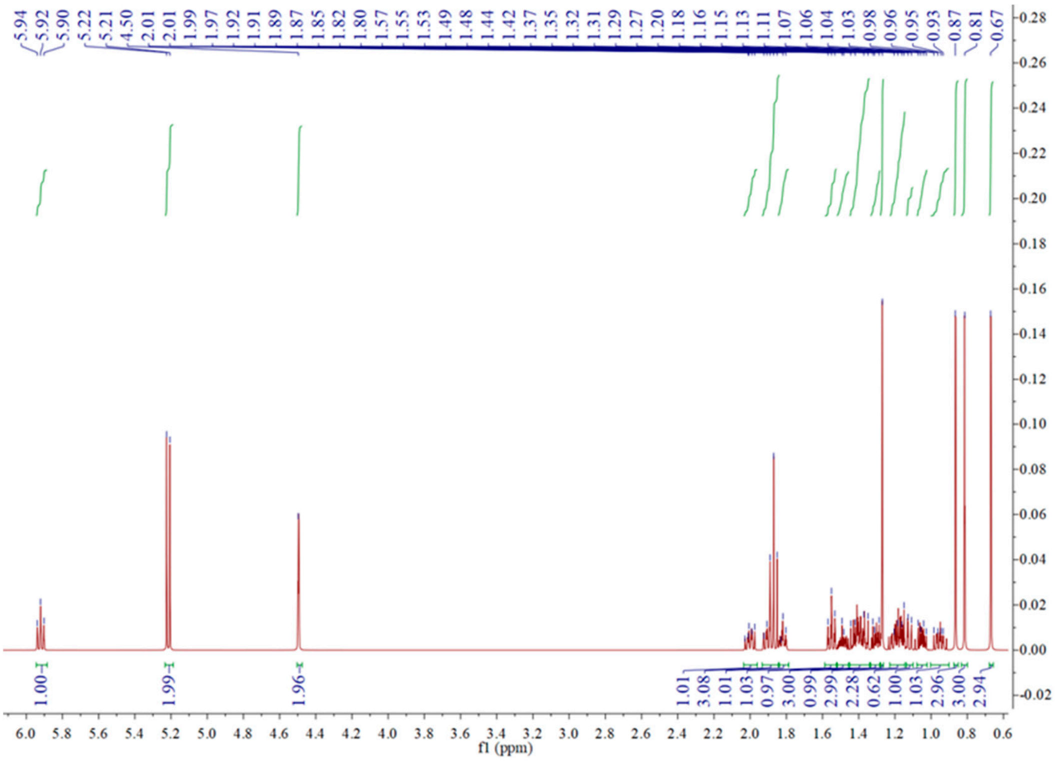

Supplementary Figure S1. ^{1}H NMR ($CDCl_3$) spectrum of compound **1**

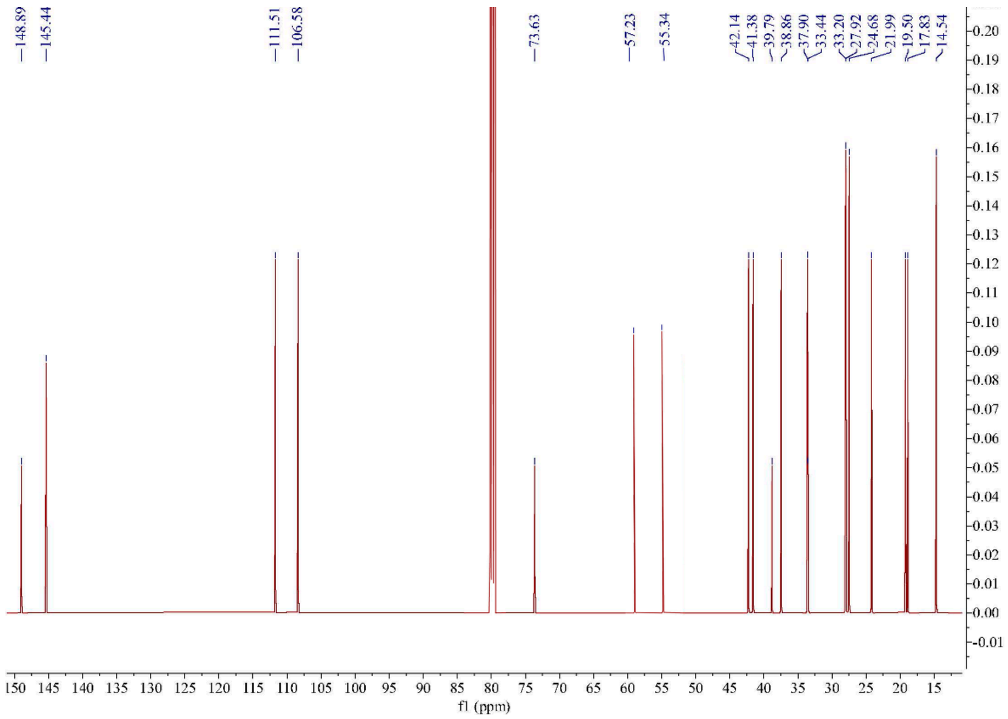

Supplementary Figure S2. ^{13}C NMR ($CDCl_3$) spectrum of compound **1**

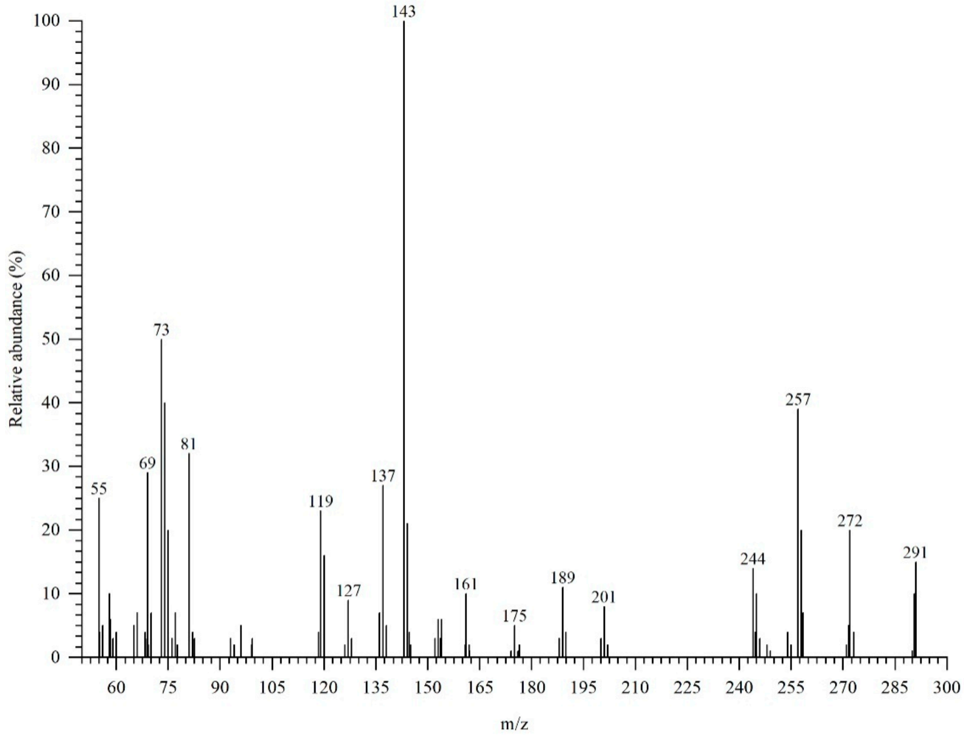

Supplementary Figure S3. ESI-MS spectrum of compound **1**

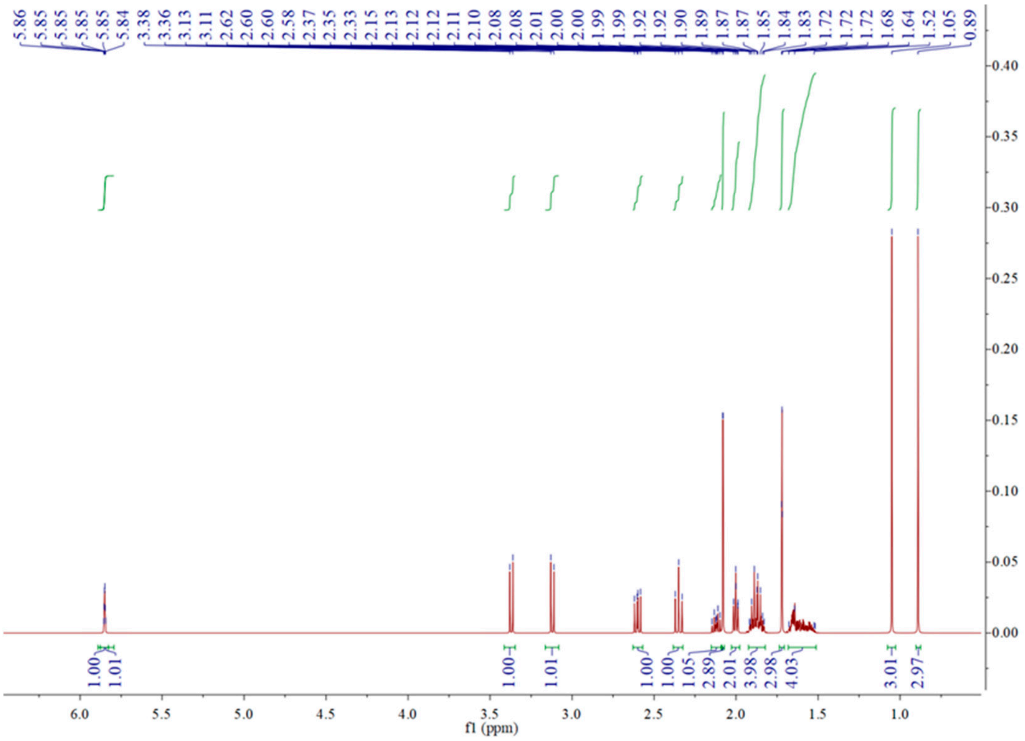

Supplementary Figure S4. ^{1}H NMR ($CDCl_3$) spectrum of compound **2**

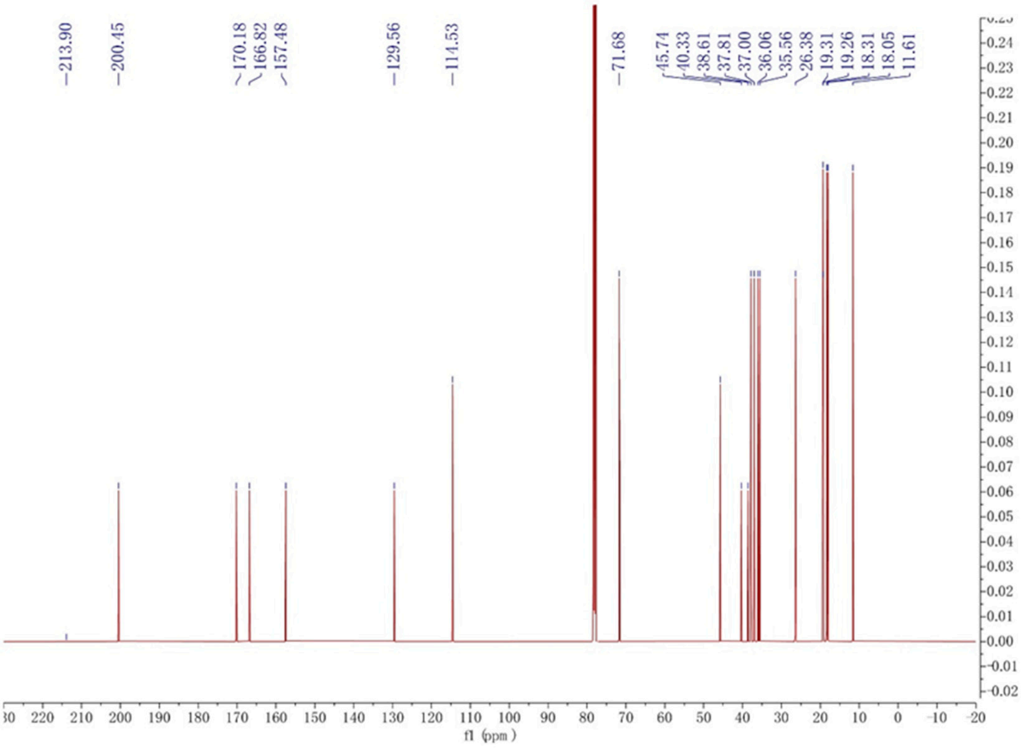

Supplementary Figure S5. ^{13}C NMR ($CDCl_3$) spectrum of compound **2**

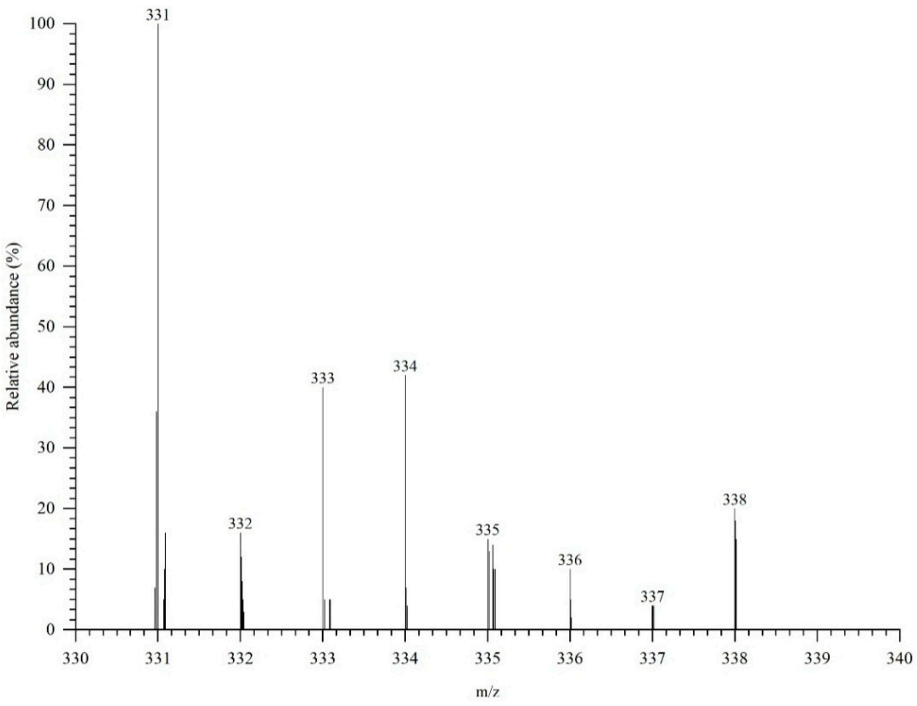

Supplementary Figure S6. ESI-MS spectrum of compound **2**

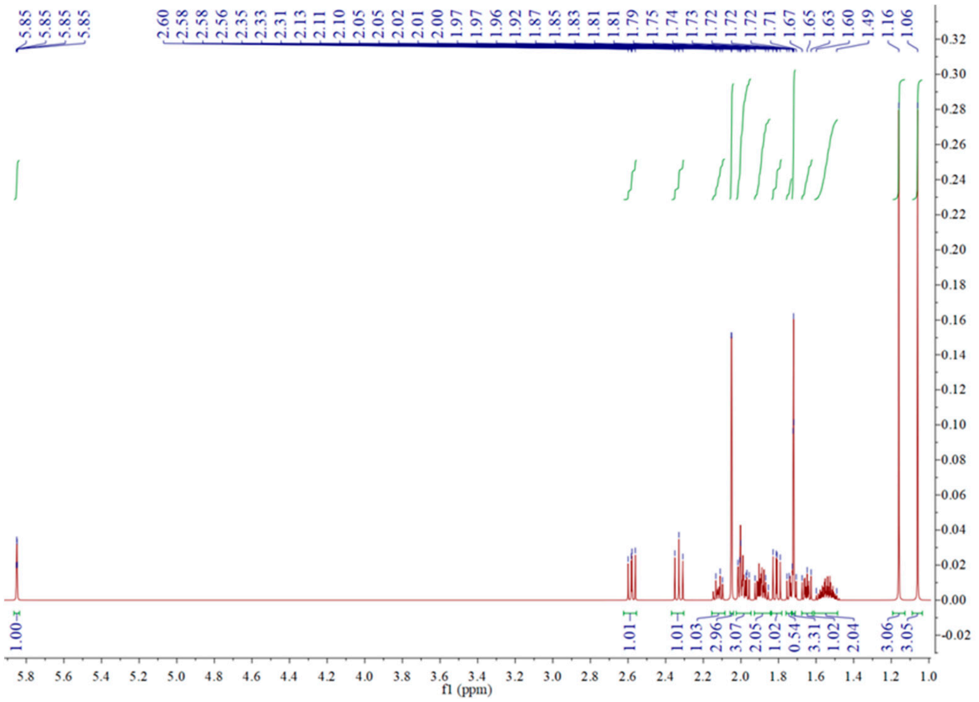

Supplementary Figure S7. ^{1}H NMR ($CDCl_3$) spectrum of compound **3**

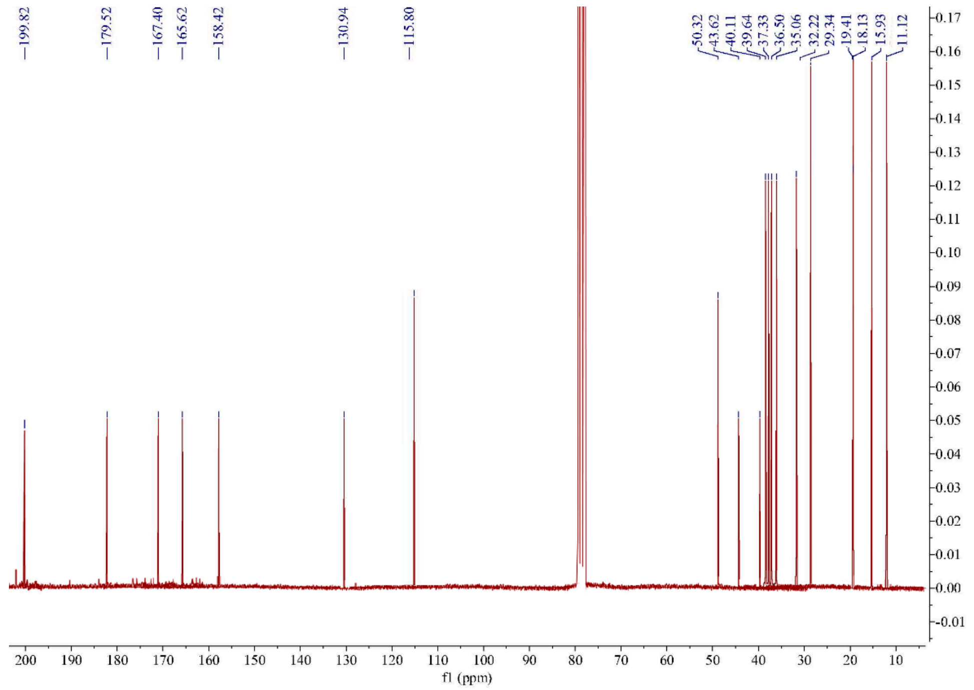

Supplementary Figure S8. ^{13}C NMR ($CDCl_3$) spectrum of compound **3**

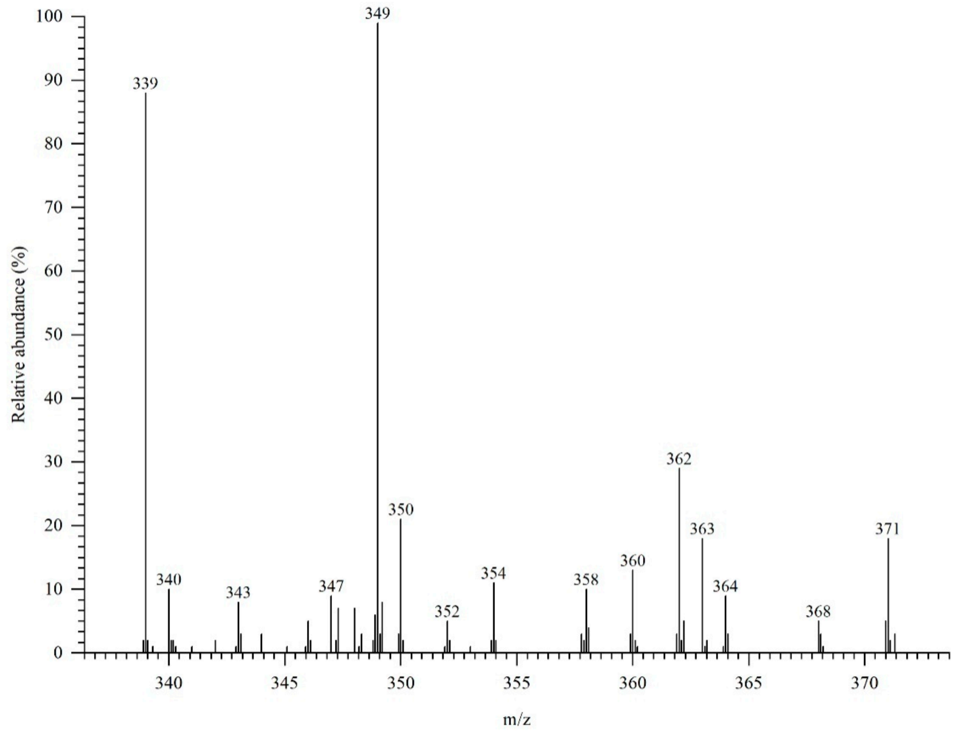

Supplementary Figure S9. ESI-MS spectrum of compound **3**

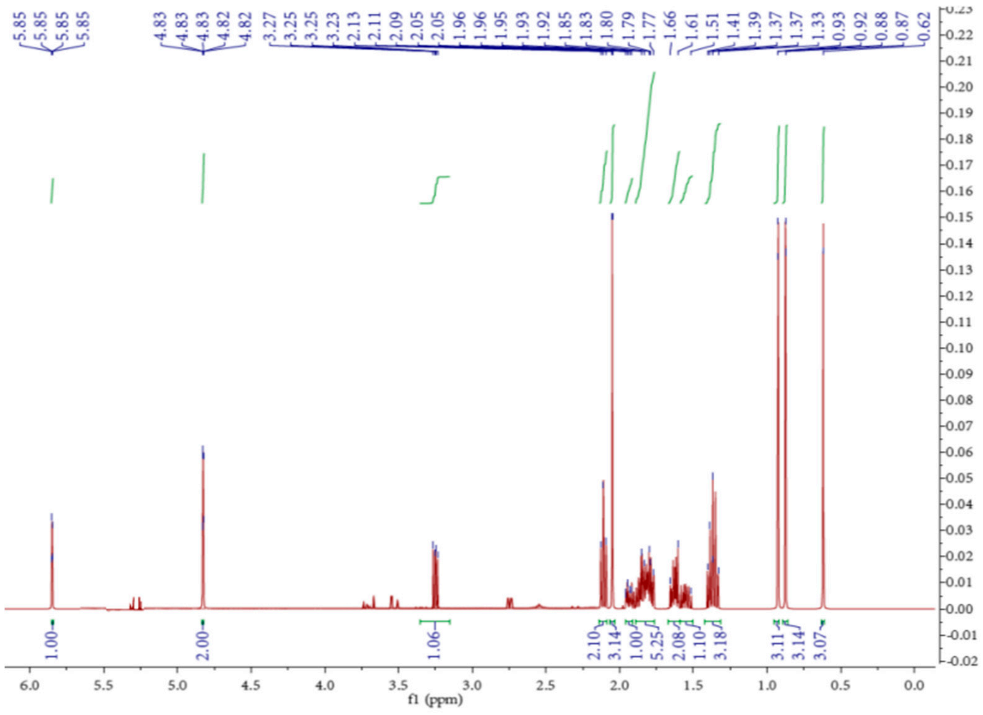

Supplementary Figure S10. ^{1}H NMR ($CDCl_3$) spectrum of compound **4**

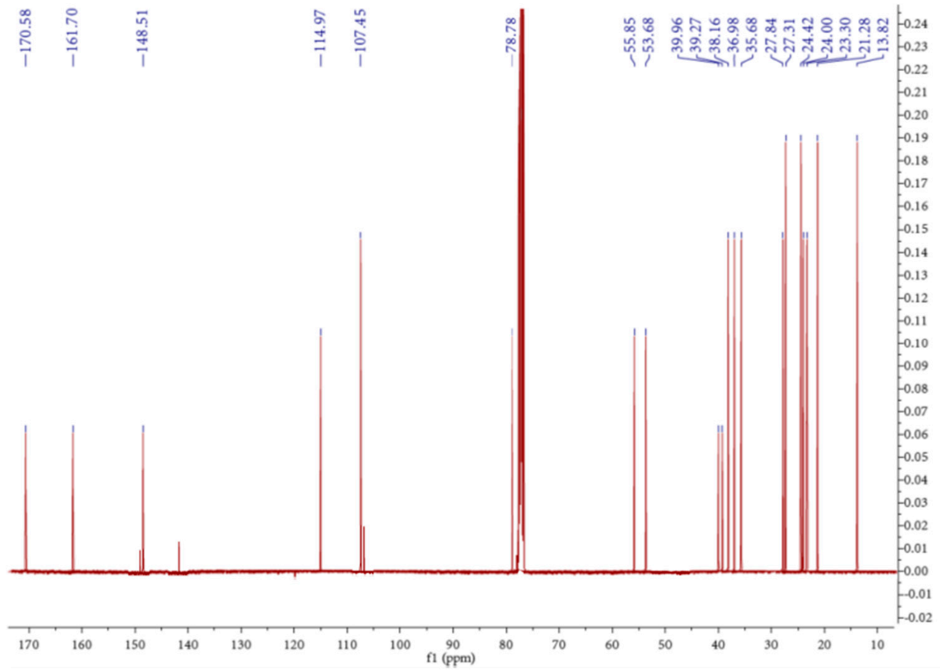

Supplementary Figure S11. ^{13}C NMR ($CDCl_3$) spectrum of compound **4**

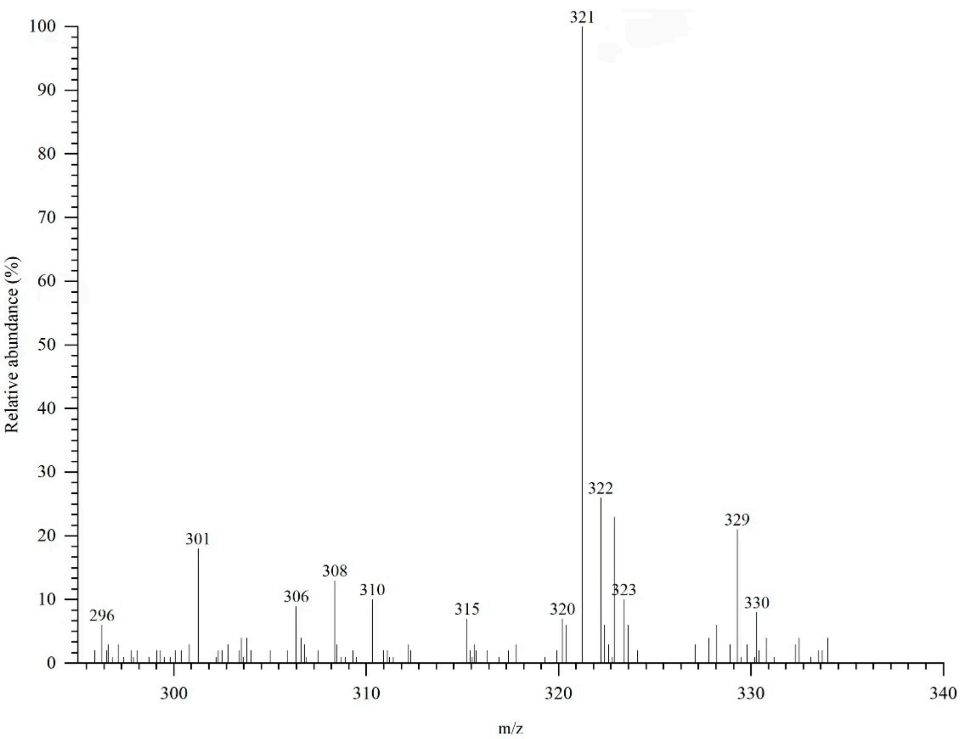

Supplementary Figure S12. ESI-MS spectrum of compound **4**

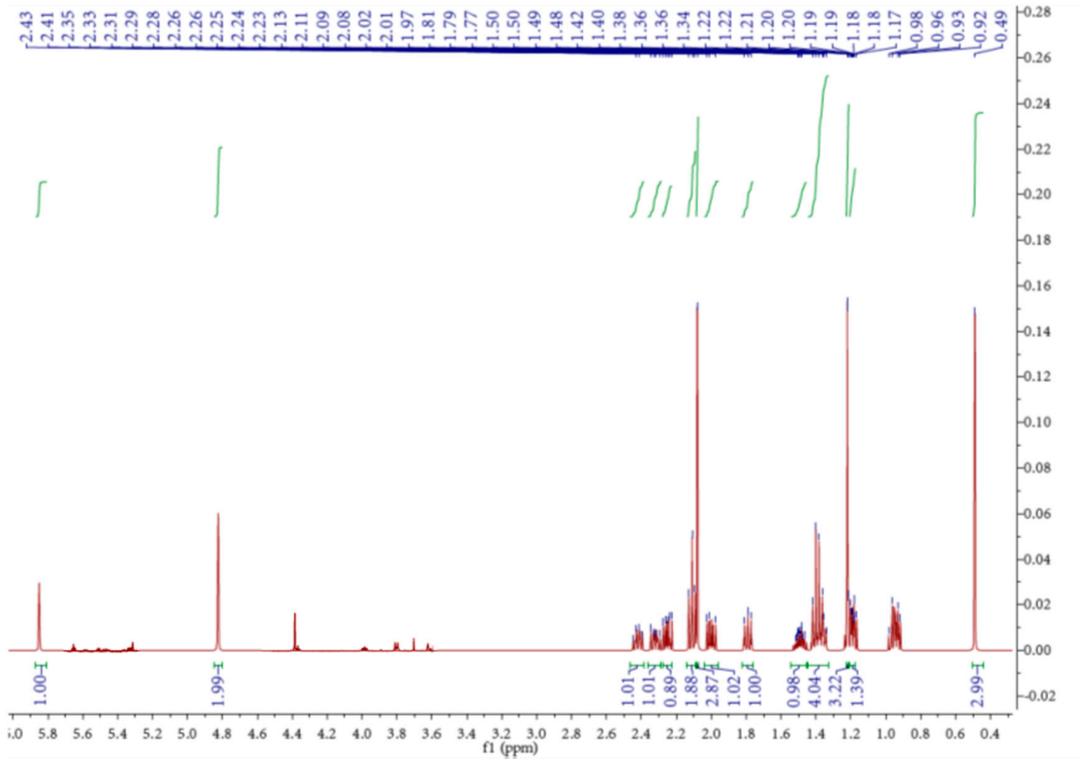

Supplementary Figure S13. ^{1}H NMR ($CDCl_3$) spectrum of compound **5**

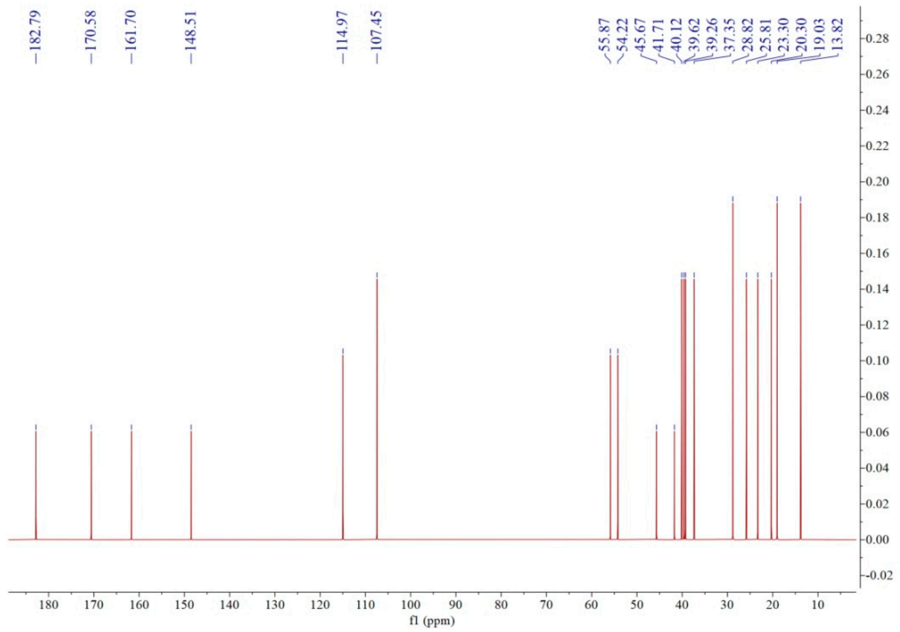

Supplementary Figure S14. ^{13}C NMR ($CDCl_3$) spectrum of compound **5**

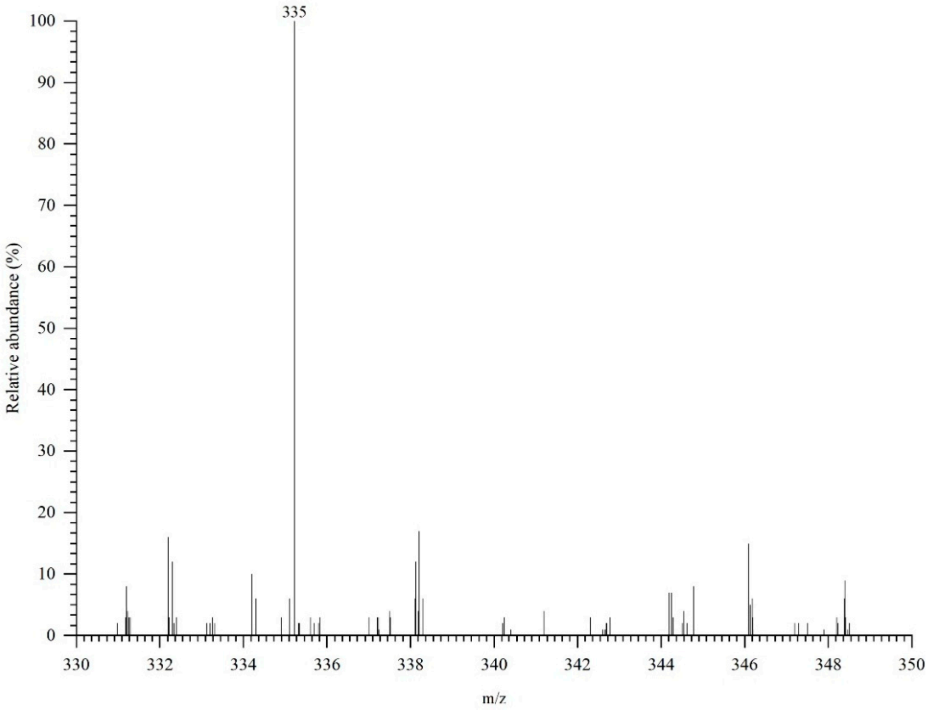

Supplementary Figure S15. ESI-MS spectrum of compound **5**

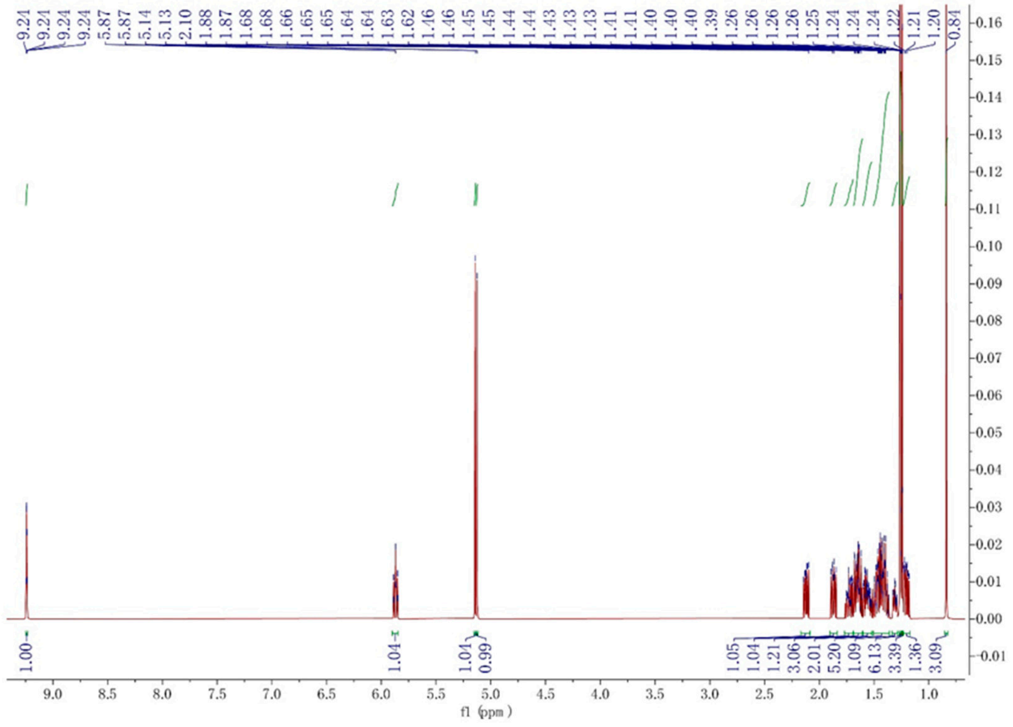

Supplementary Figure S16. ^{1}H NMR ($CDCl_3$) spectrum of compound **6**

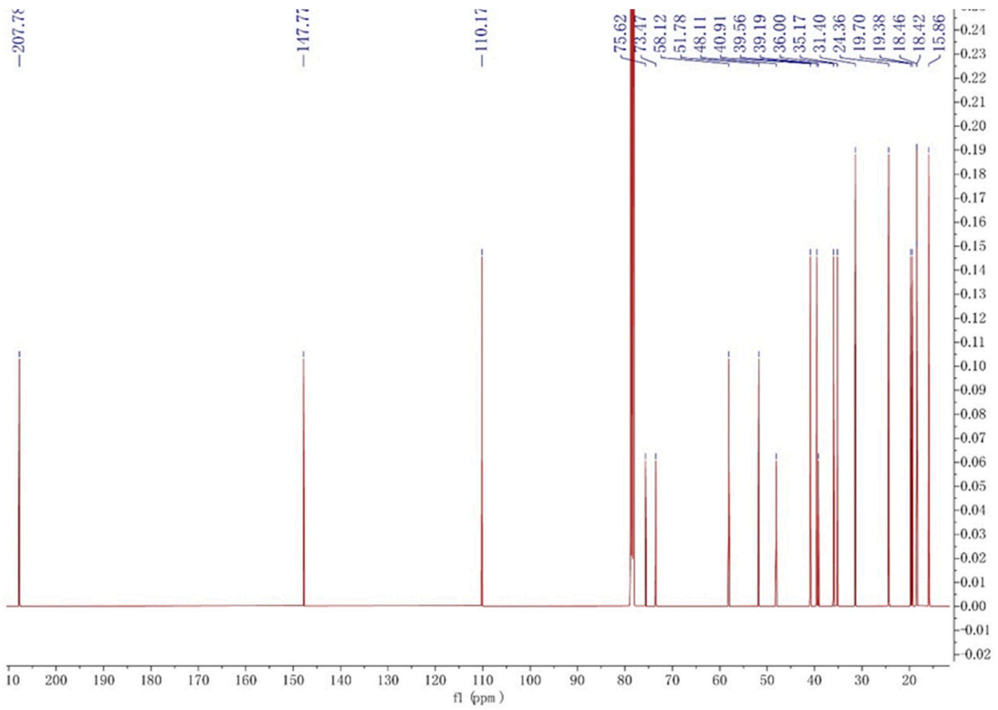

Supplementary Figure S17. ^{13}C NMR ($CDCl_3$) spectrum of compound **6**

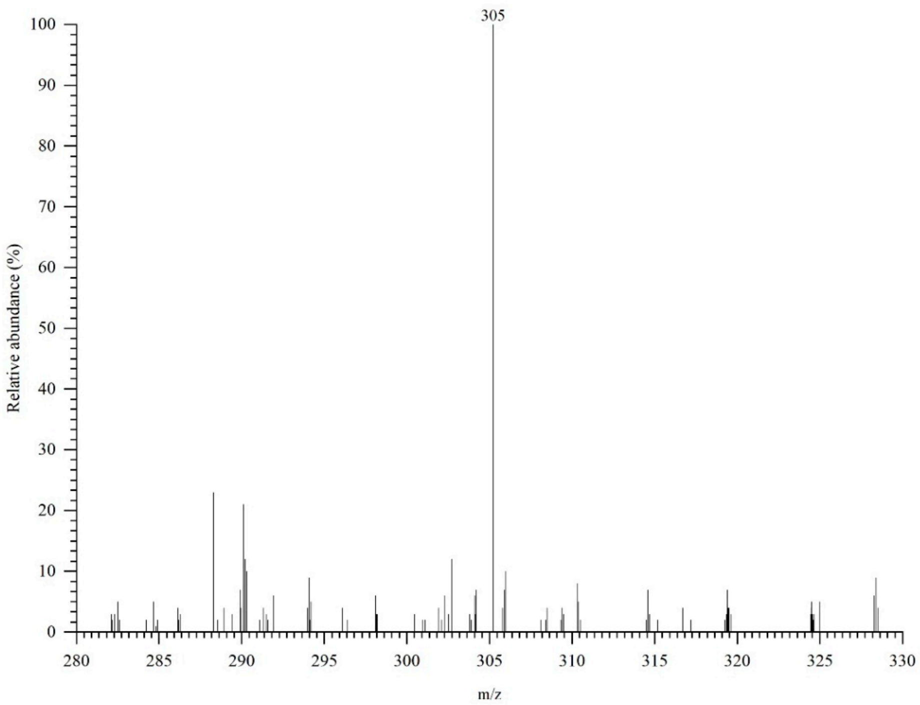

Supplementary Figure S18. ESI-MS spectrum of compound **6**

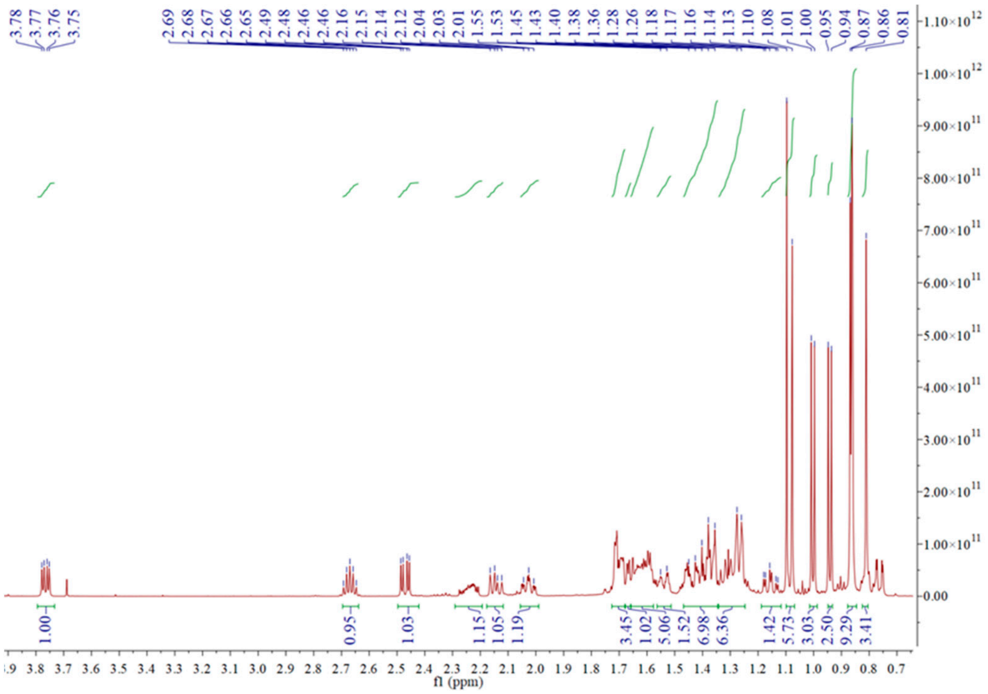

Supplementary Figure S19. ^{1}H NMR ($CDCl_3$) spectrum of compound **7**

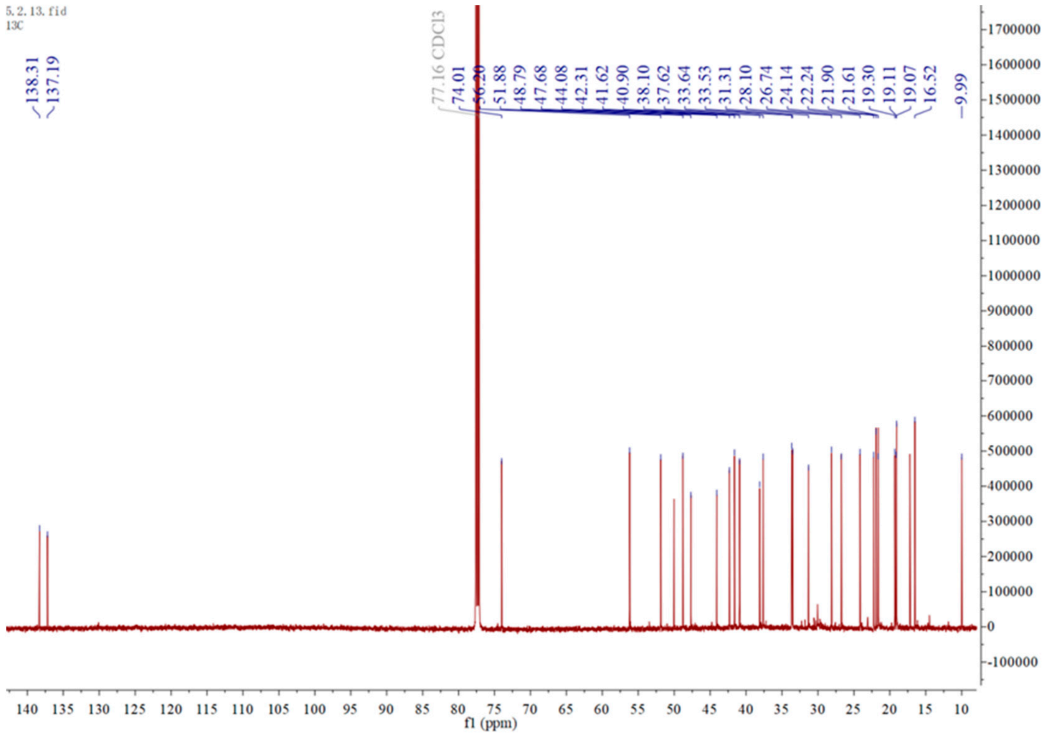

Supplementary Figure S20. ^{13}C NMR ($CDCl_3$) spectrum of compound **7**

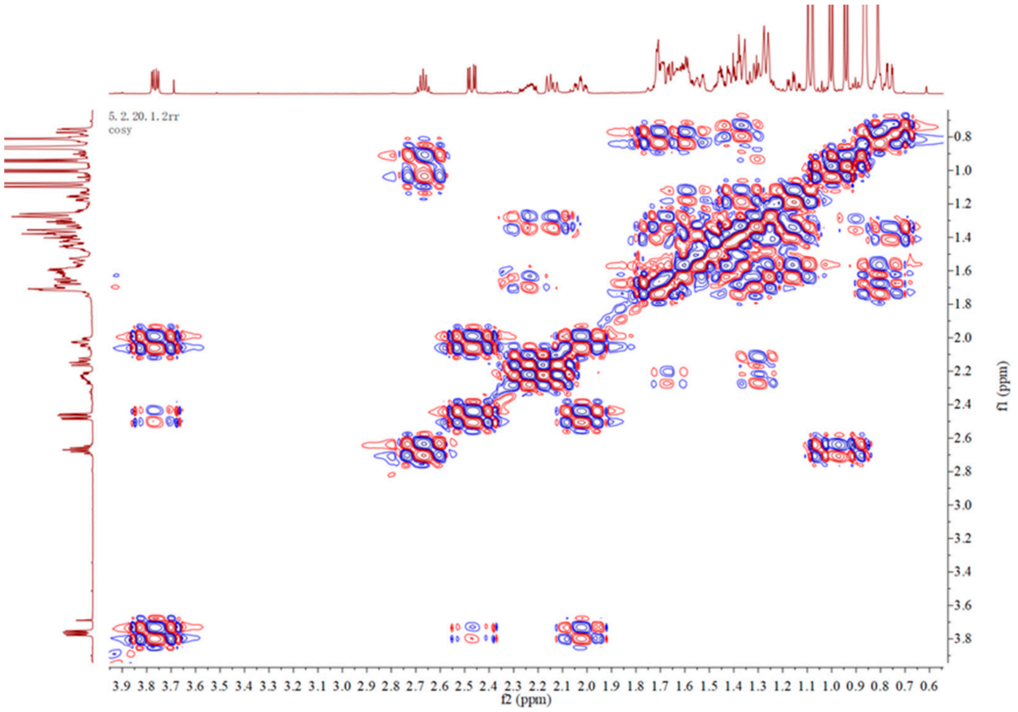

Supplementary Figure S21. COSY ($CDCl_3$) spectrum of compound **7**

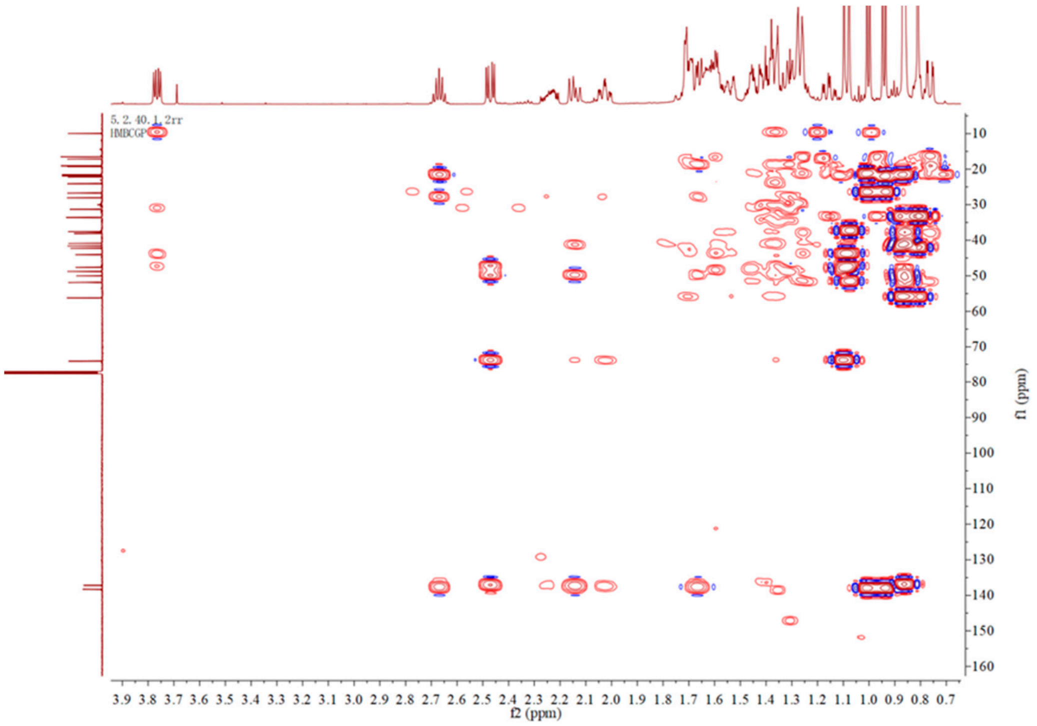

Supplementary Figure S22. HMBC ($CDCl_3$) spectrum of compound **7**

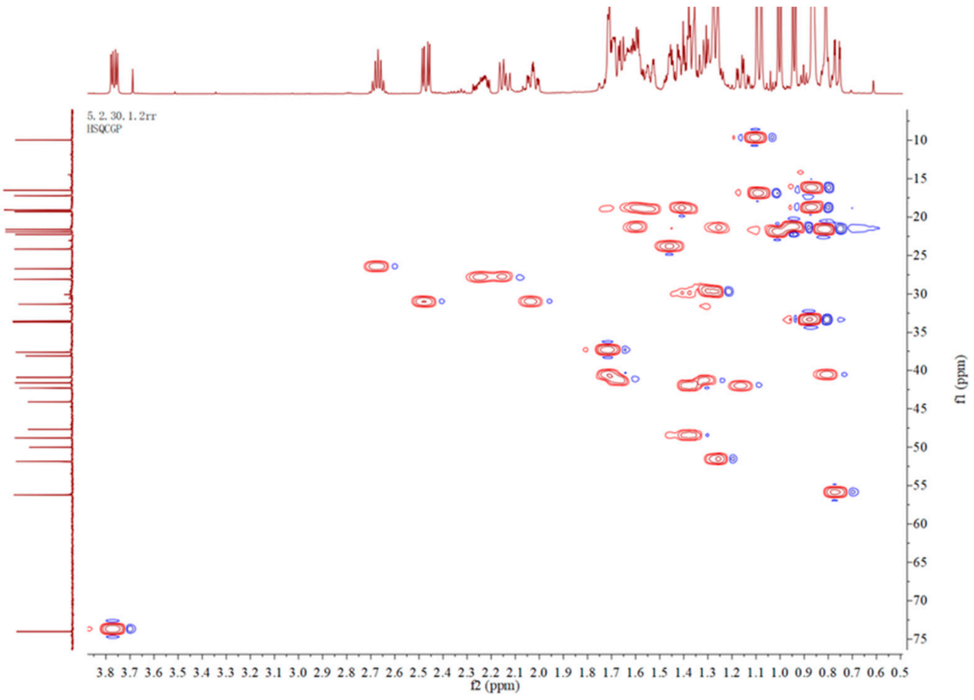

Supplementary Figure S23. HSQC ($CDCl_3$) spectrum of compound **7**

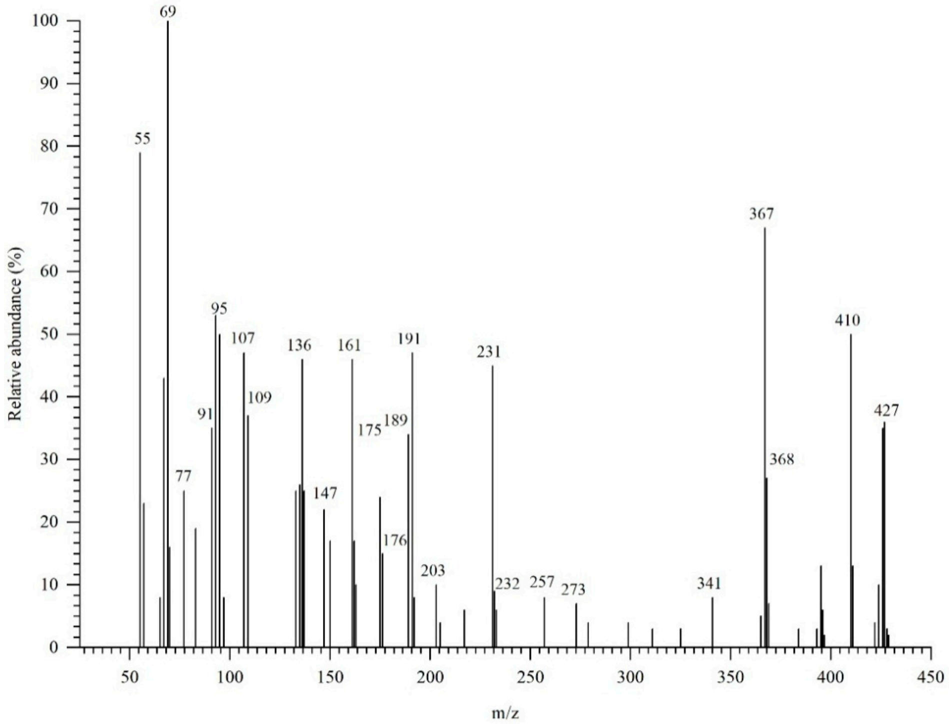

Supplementary Figure S24. ESI-MS spectrum of compound **7**

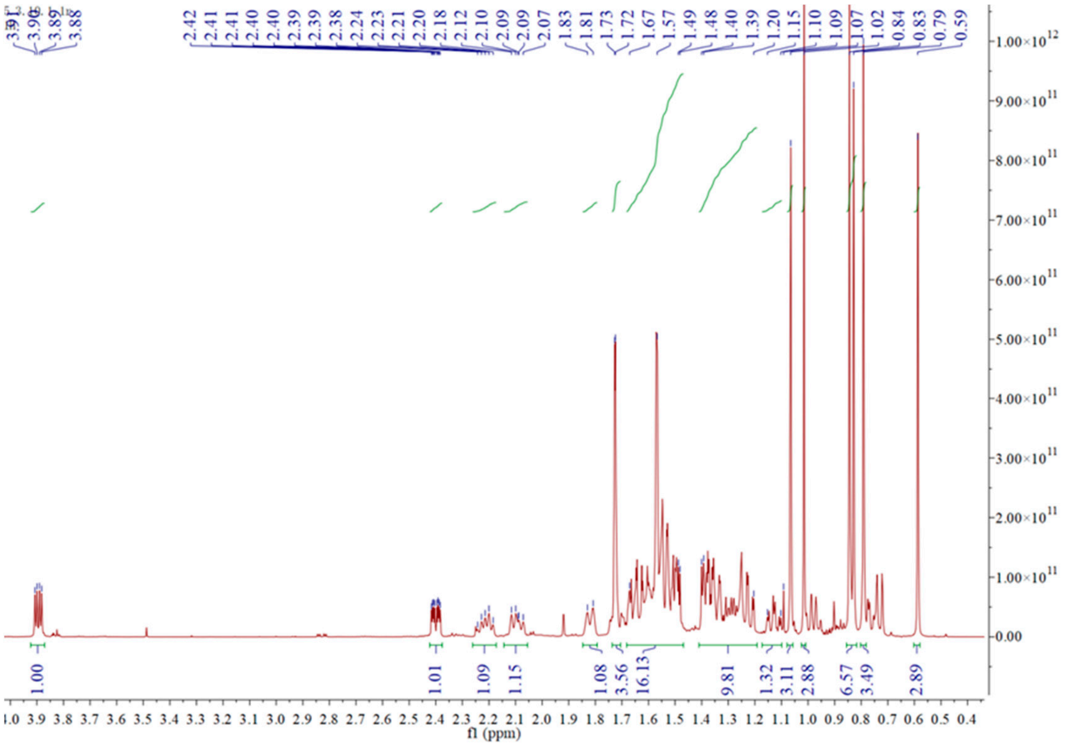

Supplementary Figure S25. ^{1}H NMR ($CDCl_3$) spectrum of compound **8**

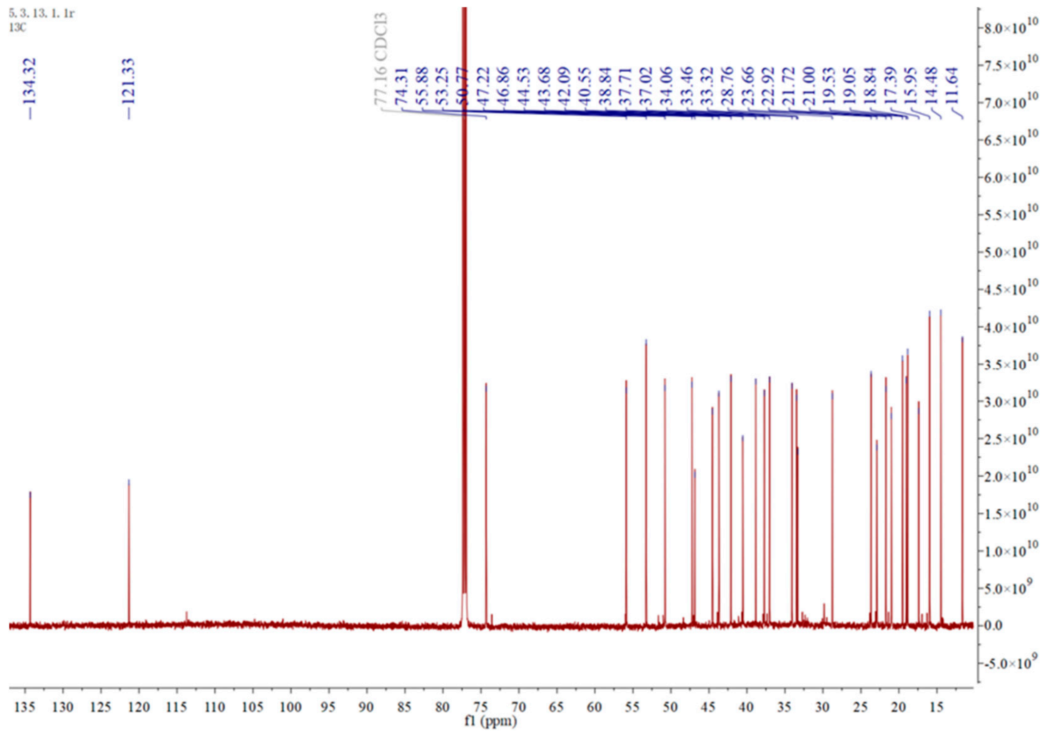

Supplementary Figure S26. ^{13}C NMR ($CDCl_3$) spectrum of compound **8**

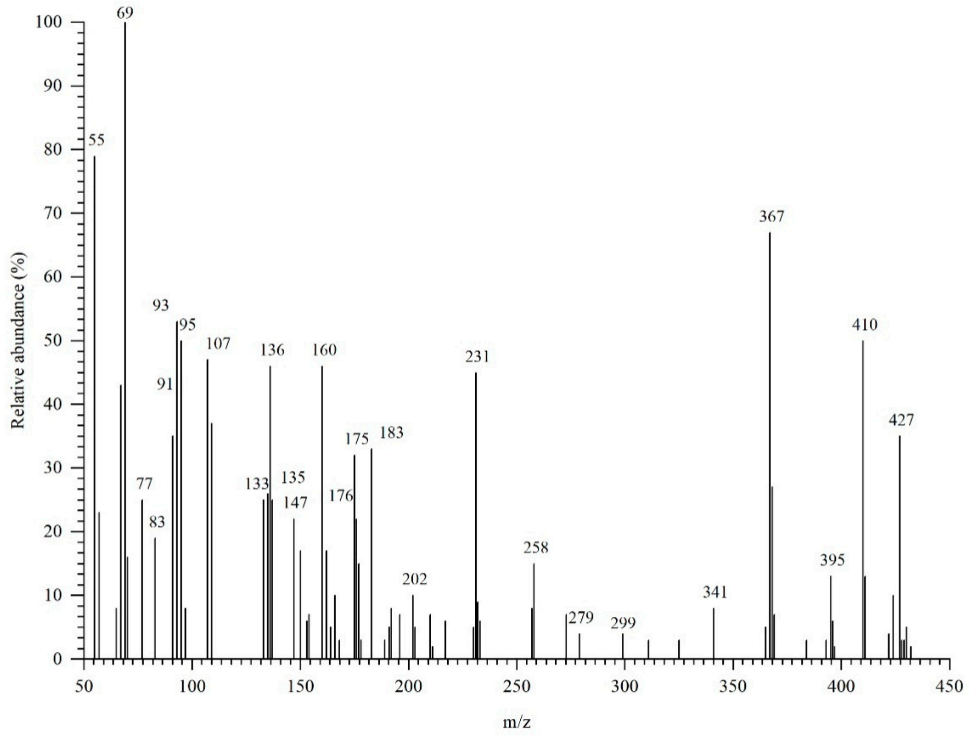

Supplementary Figure S27. ESI-MS spectrum of compound **8**

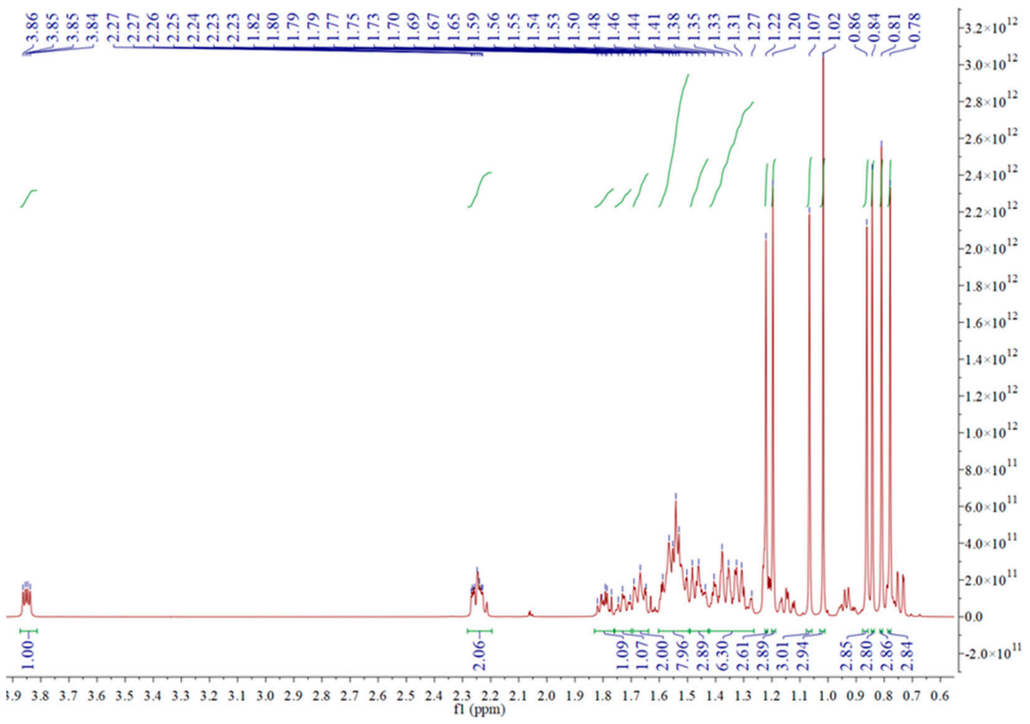

Supplementary Figure S28. ^{1}H NMR ($CDCl_3$) spectrum of compound **9**

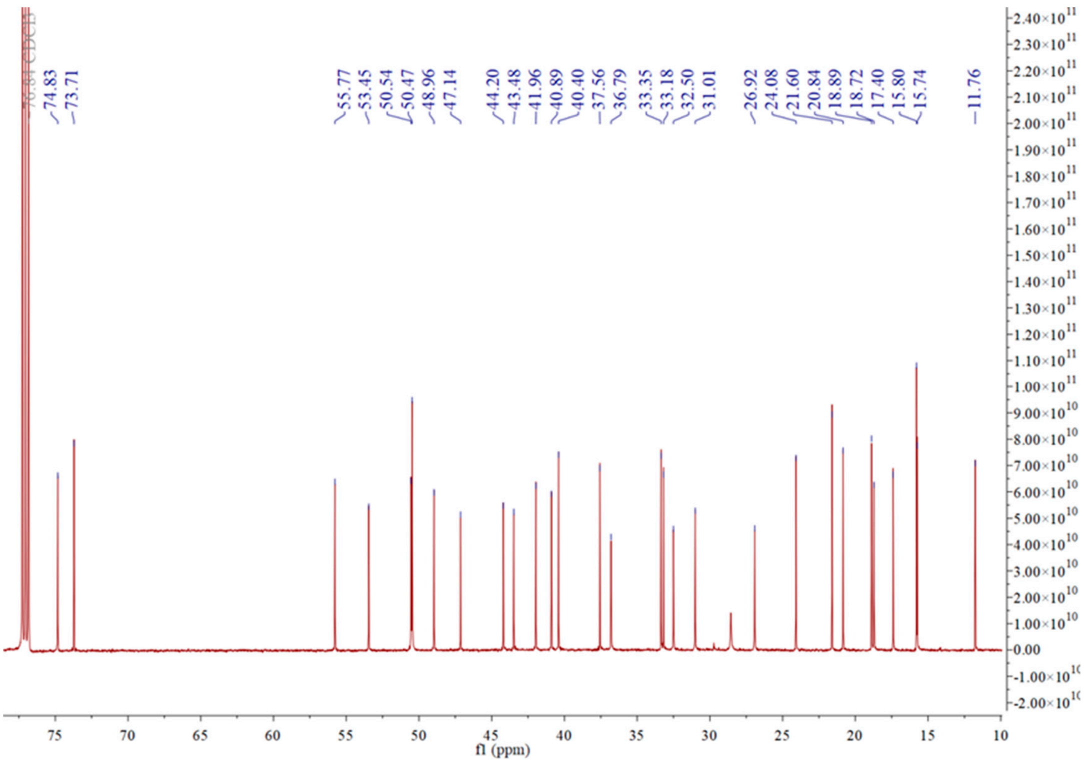

Supplementary Figure S29. ^{13}C NMR ($CDCl_3$) spectrum of compound **9**

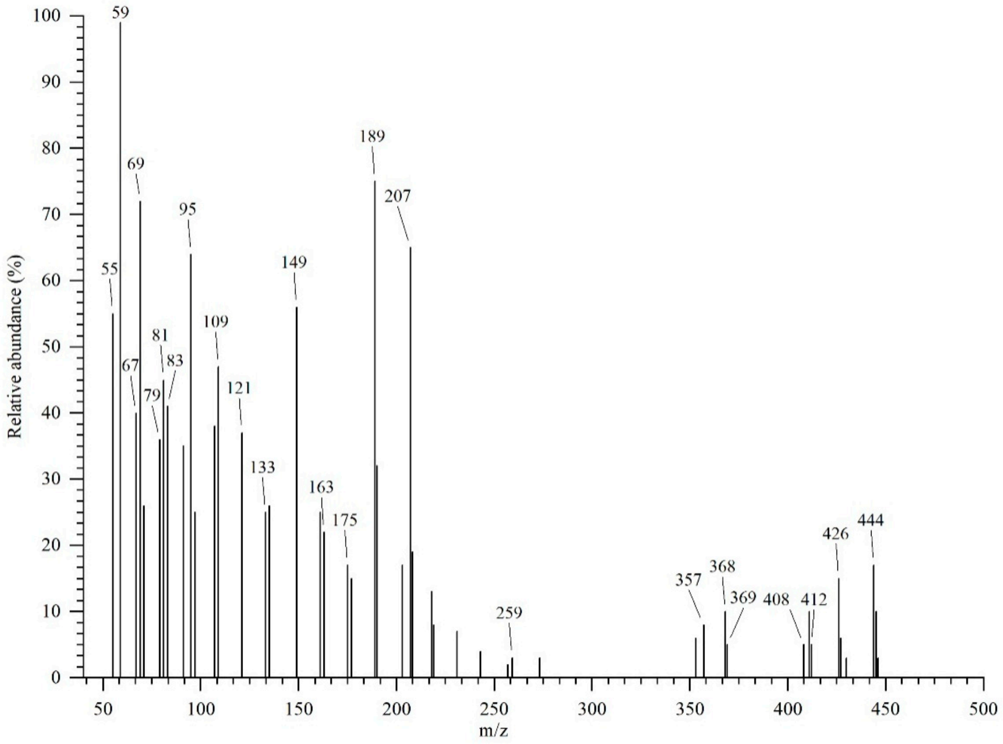

Supplementary Figure S30. ESI-MS spectrum of compound **9**

S2.2. Supplementary Tables

Supplementary Table S1. Primers used for the experiment

Primer	Primer DNA sequence
ITS1	5' -CCGTAGGTGAACCTGCGG-3'
ITS4	5' -TCCTCCGCTTATTGATATGC-3'
LR0R	5' -GTACCCGCTGAACTTAAGC-3'
LR5	5' -TTAAAAAGCTCGTAGTTGAAC-3'
T10	5' -ACGATAGGTTCACCTCCAGAC-3'
Bt2b	5' -ACCCTCAGTGTAGTGACCCTTGGC-3'

Supplementary Table S2. Species, Voucher and GenBank information of the species used in this study

Species	Voucher	ITS	LSU	*β*-Tubulin
Harringtonia aguacate	213	-	MG673961	MG674053
Harringtonia ambrosioides	18055	ON145696	ON142055	ON142055
Harringtonia ambrosioides	18056	ON145697	ON142056	ON142056
Harringtonia arthroconidialis	FLAS-F-70272	ON145695	ON142054	ON142054
Harringtonia brunnea	CBS378.68	-	EU984284	EU977460
Harringtonia chlamidospora	FLAS-F-70271	-	ON145707	ON142062
Harringtonia chlamidospora	18110	-	ON145706	ON142061
Harringtonia chlamidospora	FLAS-F-70273	-	ON145705	ON142060
Harringtonia lauricola	Raff.sp.570	MT633071	MT629759	MT644093
Harringtonia lauricola	PL159	KJ909303	-	KJ909302
Harringtonia lauricola	C2339	KF515711	-	KF515710
Harringtonia lauricola	**RL2022**	**OP893642**	**OP880432**	**OP935988**
Harringtonia sporodochialis	18073	ON145698	ON142058	ON142058
Harringtonia sporodochialis	FLAS-F-70269	-	ON142059	ON142059
Sporothrixeucalyptigena	TYPE	NR137979	NG058162	MG431426
